# From Waste to Innovation: A Circular Economy Approach for Tissue Engineering by Transforming Human Bone Waste into Novel Collagen Membranes

**DOI:** 10.3390/biom15010132

**Published:** 2025-01-15

**Authors:** Lorena Gallego, Kimberly Harvey, Marta Pevida, Luis García-Consuegra, Olivia García-Suárez, Álvaro Meana, María Alvarez-Viejo, Luis Junquera

**Affiliations:** 1 Oral and Maxillofacial Surgery Service, Cabueñes University Hospital, 33394 Gijón, Spain; lorenagallegolopez@gmail.com; 2 Department of Surgery and Specialties, Central University Hospital of Asturias, Faculty of Medicine and Health Sciences, University of Oviedo, 33011 Oviedo, Spain; garciagluis@uniovi.es; 3 Health Research Institute of the Principality of Asturias (ISPA), Foundation for Biomedical Research and Innovation in Asturias, University of Oviedo, 33011 Oviedo, Spain; marta.pevida@cruzroja.es (M.P.); garciaolivia@uniovi.es (O.G.-S.); ameana@cruzroja.es (Á.M.); alvarezvmaria@uniovi.es (M.A.-V.); 4 Biomedical Research Networking Center on Rare Diseases (CIBERER), Carlos III Health Institute (ISCIII), 28029 Madrid, Spain; 5 Tissue Engineering Unit, Asturias Community Blood and Tissue Center (CCST), 33006 Oviedo, Spain; 6 SINPOS Research Group, Department of Morphology and Cell Biology, University of Oviedo, 33006 Oviedo, Spain; 7 Unit of Cell Therapy and Regenerative Medicine, Central University Hospital of Asturias, 33011 Oviedo, Spain

**Keywords:** circular economy, bone regeneration, biodegradable polymers, collagen membrane

## Abstract

The aim of the circular economy is to treat waste as a valuable raw material, reintegrating it into the industrial economy and extending the lifecycle of subsequent products. Efforts to reduce the production of hard-to-recycle waste are becoming increasingly important to manufacturers, not only of consumer goods but also of specialized items that are difficult to manufacture, such as medical supplies, which have now become a priority for the European Union. The purpose of the study is to manufacture a novel human-purified type I collagen membrane from bone remnants typically discarded during the processing of cortico-cancellous bones in tissue banks and to evaluate its mechanical properties and effectiveness in regenerating bone-critical mandibular defects in rabbits. To prepare the novel membrane, cortico-cancellous bone chip samples from a local tissue bank were processed to isolate collagen by demineralization under agitation in HCl, cast into a silicone mold, and air-dried at room temperature and UV irradiation. The average thickness of the four batches analyzed by SEM was 37.3 μm. The average value of Young’s modulus and tensile strength obtained from the specimens was 2.56 GPa and 65.43 Mpa, respectively. The membrane’s efficacy was tested by creating a critical bicortical and bilateral osteoperiosteal defect in rabbit mandibles. The right-side defects were covered with the collagen membrane, while the left-side defects were left untreated as a control. Nine weeks post-surgery, clinical, radiological, and histological analyses demonstrated new bone formation in the treated areas, whereas the control sites showed no bone regeneration. This innovative approach not only contributes to sustainability in healthcare by optimizing biological waste but also exemplifies efficient resource use in line with the circular economy, offering a cost-effective, biocompatible option that could benefit national health systems.

## 1. Introduction

Organ and tissue transplants encompass a wide spectrum of medical interventions, ranging from life-saving procedures, such as liver transplantation, to those aimed at improving quality of life, as is frequently the case with bone grafts in patients suffering from post-traumatic nonunion, oncological or congenital disorders, or jawbone atrophy secondary to tooth loss. Certain tissues, such as bone, are utilized with minimal modification from their original state at the time of extraction from the donor [1].

Despite the critical role of bone banks, the operational framework surrounding them is not fully understood by various surgical specialists. In our region (Principality of Asturias, Spain), 373 bone tissues were collected in 2019 for processing and storage. However, a portion of this material was discarded during preparation for clinical use [2]. Alarmingly, it has been reported that today, 90% of the materials we use are wasted, lost, or are not available for reuse [3].

The aim of the circular economy is to treat waste as a valuable raw material that flows into the industrial economy, incorporating it into the lifecycle of subsequent products—specifically, the closed-loop flow of non-renewable resources. In contrast, the unsustainable management of natural resources and waste leads to adverse effects, the scale of which is often difficult to estimate [4,5]. Currently, some discarded polymeric materials have accumulated in the environment due to their high durability. Hospital waste management mainly consists of landfilling and incineration [6]. Landfills are a source of groundwater pollution, and the incineration of waste materials generates hazardous gas emissions [4]; therefore, the methodology proposed in this work can become the basic element of production within the framework of the principles of the circular economy.

One of the objectives of this work is the reuse of bone waste from a tissue bank for the manufacture of collagen membranes suitable for clinical use.

Guided bone regeneration (GBR) is an easy-to-use technique for the repair of bone defects. The GBR is based on the principle described by Dhalin et al., which describes the exclusion of inappropriate cells to allow the migration of cells that favor the osteoregeneration process [7].

The most important determinants of success in GBR procedures are the membranes used. These membranes are classified as absorbable and nonabsorbable. Among the absorbable membranes, collagen membranes (CMs) are widely used and have shown good results for over 30 years. The CMs currently used in GBR are derived from bovine and porcine sources, obtained from various structures such as the bovine Achilles tendon and internal organs such as the peritoneum, pericardium, and porcine dermal matrix [8,9].

There are so far 28 types of collagen [10], and CMs for GBR are primarily composed of type I and III collagen. These types of collagen have demonstrated the capacity for interaction and signal transduction, facilitating cell migration and proliferation within the periodontal ligament and promoting bone repair [11,12].

There are several shortcomings in current GBR technology. First, barrier failure can occur when applying membranes. One scenario involves membrane exposure to the oral environment due to poor soft tissue closure. Another situation arises when the soft tissue is well sealed, but the absorbable membrane degrades, losing its barrier protection function. Moreover, GBR largely relies on the body’s growth potential to repair defects. If the activity and number of stem cells are insufficient, predicting the osteogenic effect becomes challenging. As a result, achieving satisfactory osteogenic outcomes is difficult when the defect is large or the body is in poor condition [13].

With the rapid advancement of GBR, further improvements in barrier membranes have become one of the most important fields in bone regeneration. One approach focuses on enhancing the physical and chemical properties of membranes to strengthen their barrier function, such as through new processing methods or the preparation of multilayer structures. Another approach aims to improve osteogenic capability by incorporating drugs and various growth factors, combining osteogenesis and angiogenesis, and further reducing immune responses. By integrating growth factors into barrier membranes, GBR can significantly enhance bone regeneration [13,14].

If bone mass and stability are achieved, GBR could potentially extend beyond oral applications to improve bone augmentation in other areas. For instance, GBR might become a routine auxiliary method for repairing systemic tissue defects, such as non-benign bone defects in diabetic and osteoporotic patients, or for addressing extensive defect repairs following injuries [15].

This study aims to develop a new allogeneic collagen membrane from bone remnants produced during the processing of cortico-cancellous bones obtained from a tissue bank and to evaluate its effectiveness in regenerating bone defects in rabbit mandibles. Typically, these remnants are discarded. The study introduces a novel technique for optimizing resources and improving the efficient use of personnel and materials, consistent with the principles of the circular economy, which is crucial for national health systems.

## 2. Materials and Methods

### 2.1. Isolation and Production of Collagen I Membranes

Type I collagen was extracted from cortico-cancellous bone and processed by the tissue bank of the Community Blood and Tissue Center of the Principality of Asturias (Spain). During the preparation of bone grafts for transplantation, a portion of the tissue is discarded, and it was from this unused portion that type I collagen was obtained. Viral serology and full donor-recipient traceability were ensured for all donors in compliance with current legal regulations.

The soft tissues adhering to the bone were meticulously cleaned and washed five times with a sterile saline solution heated to 50 °C to effectively remove fat material and cellular components. Any remaining bone cells are eliminated through acid treatments to which the bone is subjected, first for decalcification and then for collagen extraction via acid hydrolysis with HCl. To initiate collagen extraction, the bone was mechanically fragmented into chips (for cancellous bone) or crushed (for cortical bone). The decalcification of the samples was achieved by incubating them in 0.5 N hydrochloric acid (HCl) for 24 h at room temperature under continuous stirring. This was followed by treatment with 1:1 acetone/methanol to eliminate lipid residues, washing with methanol to remove acetone, and two additional rinses in sterile distilled water to ensure the complete removal of methanol residues. The samples were then frozen and freeze-dried to obtain the dry weight of the product.

Collagen extraction was performed using 0.1 N HCl for five days, applying a volume of 50 mL per gram of dry tissue while maintaining constant agitation. Subsequently, the resulting mixture was centrifuged at 3000× *g* for 15 min, discarding the pellet. The supernatant was then stored at 4 °C until further use. At this stage, a measurement of type I collagen concentration was conducted using the hydroxyproline test.

For membrane production, the collagen solution (3 mg of collagen/cm^2^) was directly evaporated onto a silicone plate in a laminar flow hood over approximately 16 h. Finally, to enhance the membrane’s resistance, it was treated with ultraviolet light to promote crosslinking.

### 2.2. Morphological Analysis

The morphology and thickness of the novel membrane were examined using a scanning electron microscope (SEM, JEOL JSM-5600, JEOL Ltd., Akishima, Tokyo, Japan, acquired through JEOL’s European distribution network). A voltage of 20 kV was selected, with magnifications ranging from ×1000 to ×1500. Fixation of the membrane samples with 0.1 M glutaraldehyde and 2% phosphate-buffered saline (PBS, Gibco, London, UK) for 12 h. Followed by dehydration using a series of increasing acetone concentrations (30-50-70-90-100%). The surfaces of the analyzed samples were coated with a thin layer of gold using a BAL-TEC Sputter Coater (Model SCD 005, Leica Microsystems, Wetzlar, Germany) at a current of 19 mA and a chamber vacuum of approximately 0.03 mbar.

### 2.3. Mechanical Properties Analysis

The tensile tests were carried out using the dynamo-mechanical analysis (DMA) model RSA3 equipment from TA. The equipment has a servo-mechanical motor system and a 35 N load cell that allows both static and dynamic tests to be carried out. It also has a controlled temperature chamber, which allows tests to be carried out in conditions other than the environment. Given the capacity of the load cell, it is calibrated using a set of approved masses through the calibration application included in the equipment software (TA Orchestrator 7.2.0.4., New Castle, DE, USA). The equipment was calibrated prior to carrying out the tests. The tensile tests were carried out under ambient laboratory conditions and using a displacement speed of 1 mm/min, a speed that was considered slow enough to guarantee quasi-static test conditions. All samples were used in the dry state.

**Figure 1:**
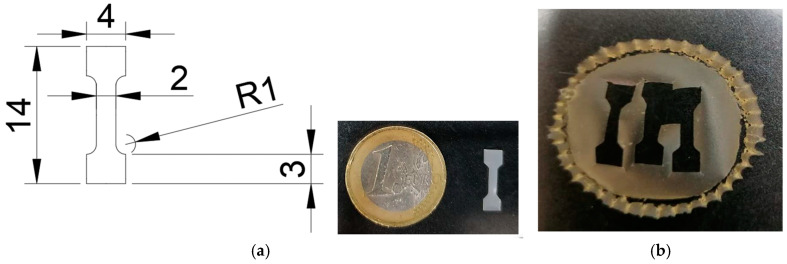
Design and dimensions of a specimen intended for mechanical tensile testing. (**a**) Technical drawing showing the geometry of the specimen, with measurements marked in millimeters. It has a central narrow section with a width of 2 mm and a radius of curvature (R1) at each transition point. The upper and lower sections are wider, measuring 4 mm, with a total height of 14 mm. This specimen’s design ensures that stress will concentrate in the narrow central section during mechanical testing. It also shows a physical specimen placed next to a one-euro coin, providing a sense of scale. (**b**) Macroscopic appearance of the membrane fabricated with the specimens used for mechanical testing.

The geometry of all the specimens was obtained using a punch, ensuring that they all had the same width (2 mm) in the investigated tensile area. The specimens featured a central region with a reduced cross-section and wider head regions, ensuring that tensile failure would occur in the central area, as is shown in Figure 1.

### 2.4. In Vivo Experiments

The study was conducted in accordance with the Declaration of Helsinki and approved by the Ethics Committee of the Principality of Asturias (protocol code CEIm number 2021.238 and date of approval 16 December 2021).

Four female New Zealand white rabbits, aged 14–15 weeks and weighing between 3.5 and 4.5 kg, were procured from Granja San Bernardo (Navarra, Spain). The rabbits were housed in the facilities of the Bioterium at the University of Oviedo (Asturias, Spain), which is registered under the number ES330440003591 with the Ministry of Rural Development and Natural Resources of the Principality of Asturias (Spain) as a breeding center and user of experimental animals, in compliance with Royal Decree 53/2013.

The animals were acclimated to their new environment for 7 days before surgery. Prior to the surgical procedures, all animals received prophylactic antibiotic therapy, and the submental area was shaved and brushed with povidone-iodine (Betadine^®^, Viatris, Madrid, Spain).

**Figure 2:**
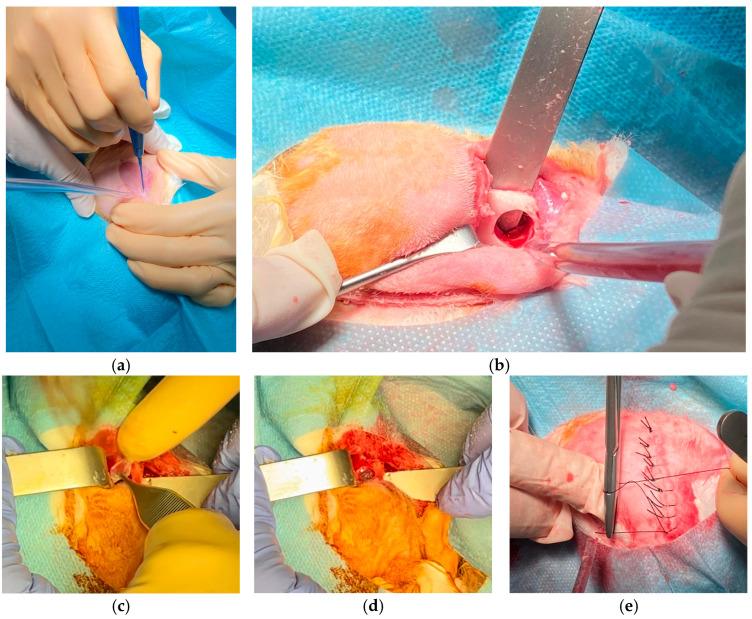
Rabbit surgery. (**a**) Mandibular approach with an incision from the chin to the midpoint between mandibular angles for bilateral mandible exposure. (**b**) Mandibular defect created using a 10 mm trephine, perforating both cortical layers without damaging soft tissue. (**c**) Placement of collagen membranes on vestibular and lingual cortices to isolate the defect. (**d**) Membranes applied without fixation, resembling ocular lenses in flexibility. (**e**) Closure of muscle and skin layers with resorbable and silk sutures, respectively.

For the surgical procedure, the animals received premedication with intramuscular morphine (3 mg/kg) and subcutaneous meloxicam (0.3 mg/kg). General anesthesia was then induced via intravenous injection of medetomidine (0.12 mg/kg) and midazolam (2 mg/kg). Following this, a bilateral exposure of the mandible was achieved through an incision extending from the chin to the midpoint between the left and right mandibular angles (Figure 2a). The mandibular vestibular cortex was subsequently exposed bilaterally. To create the mandibular defect, a 10 mm trephine was employed, ensuring that the vestibular and lingual cortices were evenly perforated while avoiding injury to the soft tissue on the lingual surface and the basal bone (Figure 2b). The collagen membranes were placed on the vestibular and lingual cortical (Figure 2c) of the right side of the animals, thereby isolating the defect generated. The membranes were not secured with any type of glue, and their mechanical behavior resembled that of an ocular lens (Figure 2d). On the left side of the animals (control side), the defect created remained membrane-free. Finally, the muscle over the defect was closed using loose resorbable sutures (Vicryl 4-0), and the skin was sutured with loose silk sutures (4-0) (Figure 2e).

After the surgery, the rabbits were kept on a soft diet until euthanasia. All animals were euthanized nine weeks postoperatively.

### 2.5. Computed Tomography (CT) and Micro-CT Analyses (μCT)

**Figure 3:**
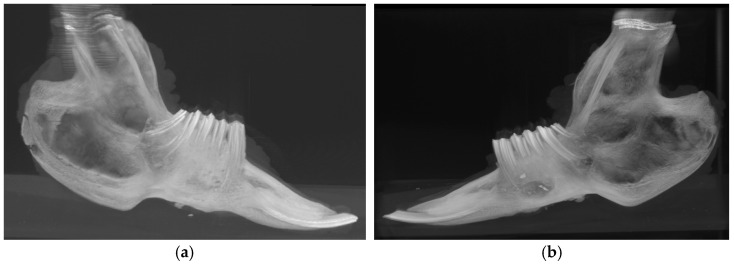
Computed tomography image of complete jaws. (**a**) Bone regeneration observed on the right side of the animal, where the defect was covered with a membrane. (**b**) Persistent defect on the left side of the animal (control side), where no membrane was applied.

The complete jaws (Figure 3) of the four rabbits euthanized at the conclusion of the nine-week experiment were analyzed immediately post-euthanasia using computed tomography (CT) (ARGUS PET-CT, Sedecal, Spain). The imaging was conducted with the following operational parameters: a small target, 120 kV, 125 mA, a scanning time of 1 s, and a section thickness of 0.5 mm. Additional specifications included a current of 300 µA, a voltage of 45 kV, 1080 projections, 4 shots, high resolution, and a total scanning duration of 25 min and 30 s. Multiplanar and three-dimensional DICOM images were subsequently generated using Preclinica Uniovi Fiji 1.11 image processing software (Universidad de Oviedo, Spain).

For micro-computed tomography (μCT) analysis, a 20 × 15 mm bone segment was removed from the right mandibular body. Two areas of interest were determined for each experimental right hemimandible: native bone (1.5 mm surrounding the surgical defect) and new-formed bone. A qualitative analysis of the total mineral and newly formed bone contents was performed using a high-resolution micro-CT system (SkyScan 1174, SkyScan, Kontich, Belgium) and subjected to 3D reconstruction using NRecon software (NRecon software version 2.0, Bruker, Billerica, MA, USA). The scanner was equipped with a 20 to 100 kV (10 W) X-ray source and an 11-megapixel X-ray detector.

Each sample was placed on a holder with the sagittal suture oriented parallel to the X-ray detector and scanned using a 0.11 mm copper filter, 26 μm isotropic voxels, a 0.9 rotation step, and frame averaging of 2. The following variables were studied: Bone Mineral Density (BMD), expressed in grams of hydroxyapatite per cubic centimeter (gHA/cm^3^), Bone Volume/Total Volume (BV/TV), defined as the ratio of the segmented bone volume to the total volume of the region of interest, measured as a percentage, Trabecular Thickness (TbTh), expressed in mm, Trabecular Number (TbN), expressed as 1/mm and Trabecular Separation (TbSp) expressed in mm.

### 2.6. Histological Examination

Macroscopically, the following parameters were evaluated: (a) anatomical and tissue organization of the defect, (b) infections, (c) biomaterial displacement or extrusion, (d) bone sequestration, and (e) consistency and morphology of the defect.

After radiological examination, mandibular transversal sections of approximately 2–3 mm in thickness were taken from the anterior, central, and posterior areas of each regenerated bone and decalcified in buffered 10% formaldehyde supplemented with 0.7% nitric acid for 4–7 days. Once decalcified, sections were washed with distilled water, dehydrated in a graded series of ethanol dilutions, and embedded in paraffin. Sections of 5–7 μm thickness of each sample were obtained using a microtome HM 350 S (Microm, Waldof, Germany). These sections were stained with Hematoxylin-eosin (H&E), Von Kossa, Masson’s trichrome, or Vimentin and analyzed in depth.

### 2.7. Statistical Analysis

All statistical calculations were performed using SPSS software 27.0.1. (SPSS Inc., Chicago, IL, USA). Quantitative micro-CT parameters are expressed as mean ± standard deviation. After testing for normality and equal variance, differences between native and new bone were analyzed by one-way analysis of variance (ANOVA) followed by the Bonferrony multiple comparison test. Differences were considered significant when *p* < 0.05, based on the values obtained in micro-CT analysis.

## 3. Results

### 3.1. Morphological Observation

**Figure 4:**
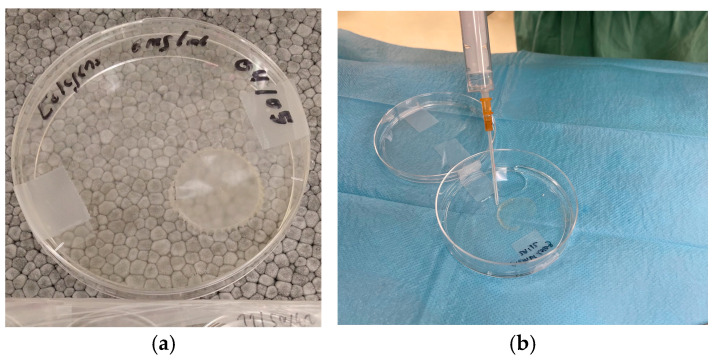
Macroscopic appearance of the membrane fabricated. (**a**) Newly fabricated membrane showing a whitish-transparent color. (**b**) Hydration of the membrane with physiological saline; as shown, it acquires a transparent appearance similar to that of a contact lens.

All collagen membranes obtained by directly evaporating purified collagen I onto a silicone plate were mostly transparent, with a uniform diameter and thickness (Figure 4).

It is shown in Table 1 the thickness values obtained for each of the four membrane batches analyzed by SEM. For each membrane analyzed, three different images and five measurements on each were taken. Within batches 1–3, the membrane thickness was acceptably homogeneous. However, in batch 4 the thicknesses were almost 50% lower than in the other batches. Additionally, in batches 1 and 2, there is a tendency for the thickness at the edges (in table: excess material) to be lower than in the central (in table: specimen) area of the sample.

**Figure 5:**
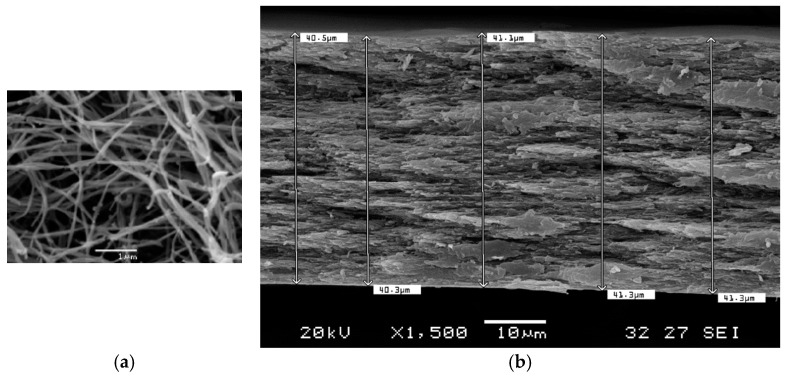
Scanning electron microscope image of the fabricated membranes. (**a**) showing the fibrous structure collagen membrane. (**b**) Example of thickness measurement of the samples.

Figure 5 shows an image obtained by scanning electron microscopy of one of the fabricated membranes. It can be observed (Figure 5a) that the membrane is primarily composed of type I collagen, as evidenced by the ring-like morphology of the triple-helix fibers irregularly intertwined, along with an unidentified component, as the color variation in the image indicates a difference in material density.

Meanwhile, Figure 5b provides a more insightful cross-sectional view of the collagen membrane, showing the internal layering and structural density of the sample. This view reveals a stratified, compact arrangement of collagen fibers, giving the membrane a dense, lamellar appearance. Each layer exhibits a rough texture, which suggests the fibrous and layered nature typical of collagen membranes. This multi-layered architecture likely enhances mechanical stability while retaining some porosity. The dense areas would contribute to tensile strength, enabling effective support under physiological conditions while allowing nutrient and cellular movement within the structure, which is essential in the GBR process.

### 3.2. Mechanical Properties

Tensile tests were performed under laboratory ambient conditions, using a displacement rate of 1 mm/min. Applying stress (force per unit area) as it deforms (strain) in percentage terms. The average thickness values for each batch, as presented in Table 1, were used as the thickness measurement for each specimen.

**Figure 6:**
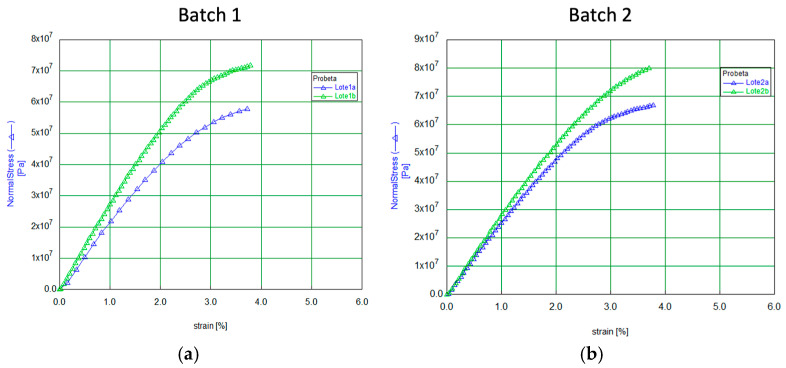
Stress-strain curves for the specimens from batches (**a**) 1 and (**b**) 2.

**Figure 7:**
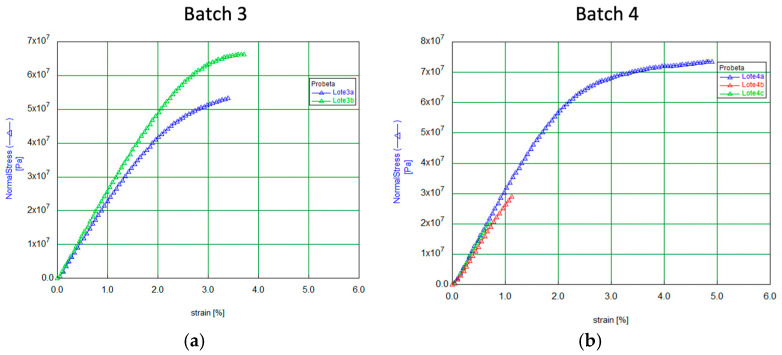
Stress-strain curves for the specimens from batches (**a**) 3 and (**b**) 4.

Figure 6 and Figure 7 present the tensile curves (stress-strain) corresponding to the specimens extracted from the different batches. From each membrane, only two specimens were taken from the most central area of the sample, except for one of them (batch 4), from which three specimens were extracted.

The curves demonstrate that all specimens exhibit an initial linear region, where stress increases proportionally with strain, suggesting an elastic response up to approximately 1% strain, followed by a non-linear plastic behavior, indicating that the material is reaching its maximum stress tolerance. Again, batch 4 presents a different behavior. Significantly, this batch is also the one with the smallest thickness (almost 50% less than the others).

In our study, the so-called Young’s modulus (*E*), which describes the stress-strain relationship, was calculated by applying a linear fit to the initial elastic region of the curve, up to 1% deformation. The relationship, expressed as *σ* = *Eε*, is shown in Table 2, where *σ* represents the stress and *ε* represents the strain. This table also includes the tensile strength values (maximum values of the curves) for each specimen.

It is a resume in Table 3 the average values of the mechanical parameters that can be considered representative of the fabricated membranes (Batches 1–3).

### 3.3. Radiological and Micro-CT Analyses (μCT)

**Figure 8:**
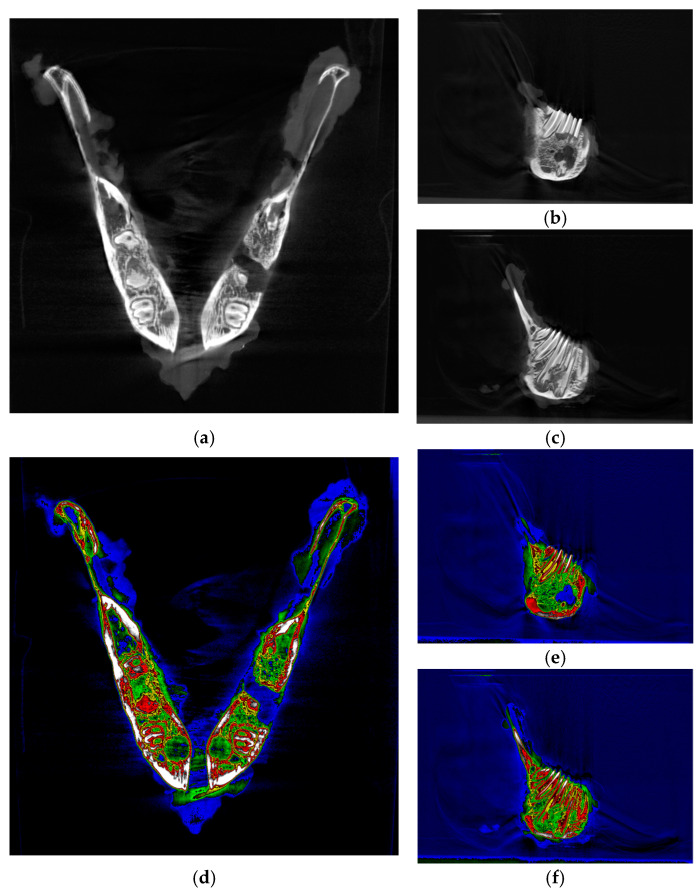
Computed tomography image of complete jaws. Image resolution: 31.87 µm/pixel. No magnification was applied. (**a**) Axial CT view highlighting anatomical regions with a defect observed on the left side and evidence of regeneration on the right. (**b**) Sagittal CT view with a persistent defect on the left side of the animal (control side), where no membrane was applied. (**c**) Sagittal CT view with bone regeneration on the right side of the animal, where the defect was covered with a membrane. (**d**–**f**) Same images as from (**a**–**c**) but with a color palette representing different bone densities, where blue indicates the lowest density (hypodensity).

The PET-CT images demonstrate a marked increase in bone neoformation in the experimental hemimandible (right side) compared to the control side, where no bone formation was observed in any animal (Figure 8). However, radiographically, complete mineralization of the experimental defect could not be observed in any of the four rabbits.

**Figure 9:**
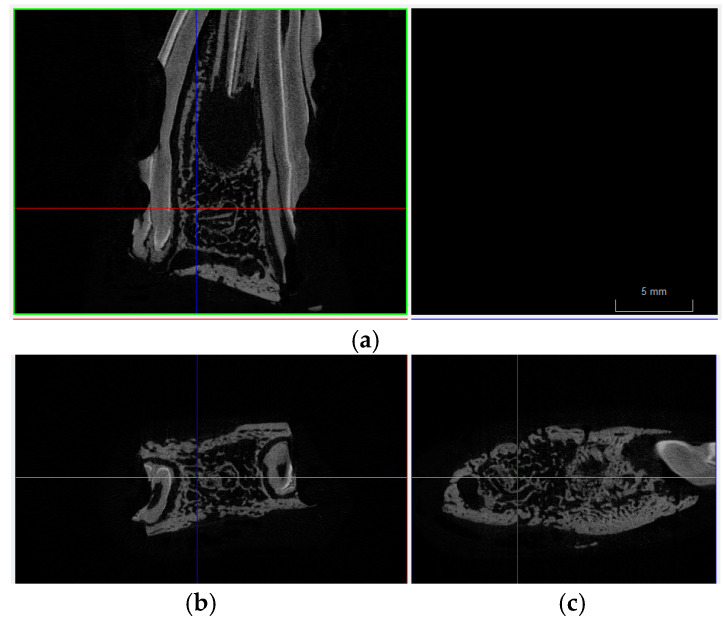
Multiple cross-sectional micro-CT views of a bone regeneration site. (**a**) longitudinal view, capturing both cortical and trabecular bone regions. (**b**) axial and (**c**) sagittal perspectives of the regenerated bone matrix, providing detailed insight into the bone formation and structural integration within the zone.

Micro-CT images confirm bone regeneration, as shown in Figure 9, revealing the internal integrity structure and density contrasts within the area.

Table 4 presents the descriptive values for native and newly formed bone (cortical and trabecular) across the five variables investigated after 9 weeks of grafting.

The differences between native and newly formed bone were significant in all variables studied, except for trabecular thickness. In the post-hoc test, these differences always occurred between native and newly formed trabecular bone, never between native and cortical bone. The newly formed bone also exhibited a reduced number of trabeculae, with greater spacing between them but with the same thickness as those present in the native bone.

**Figure 10:**
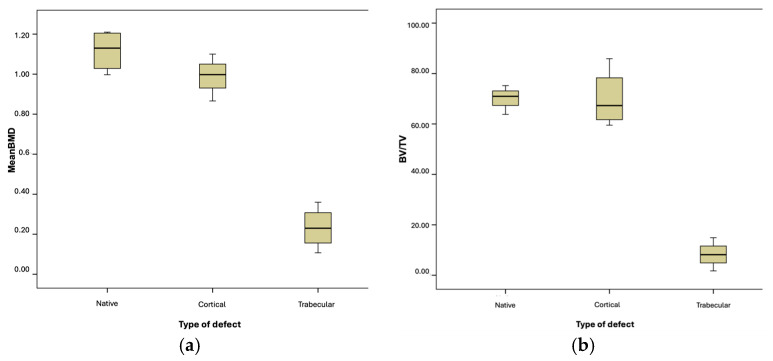
(**a**) Mean BMD variable. (**b**) BV/TV ratio.

Figure 10 illustrate the significant differences between native and newly formed trabecular bone in the variables BMD and BV/TV.

**Figure 11:**
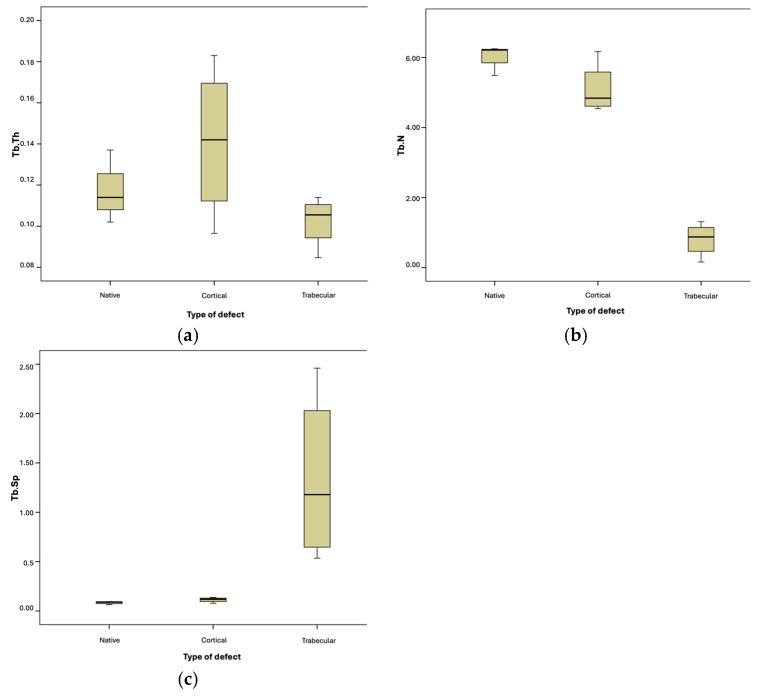
Box-and-whisker plot graphic of trabecular bone comparison by type of defect. (**a**) Trabecular Thickness. (**b**) Trabecular Number. (**c**) Trabecular Separation.

The differences between native and trabecular bone were also significant for the variables TbN and TbSp. However, they were not significant for the variable TbTh (Figure 11).

In summary, the use of the new membranes in the present study demonstrated a higher degree of mineralization and new bone formation than the non-grafted control defects. However, compared to the native bone adjacent to the defect, the amount of bone and the degree of mineralization were significantly lower.

### 3.4. Histological Observation

**Figure 12:**
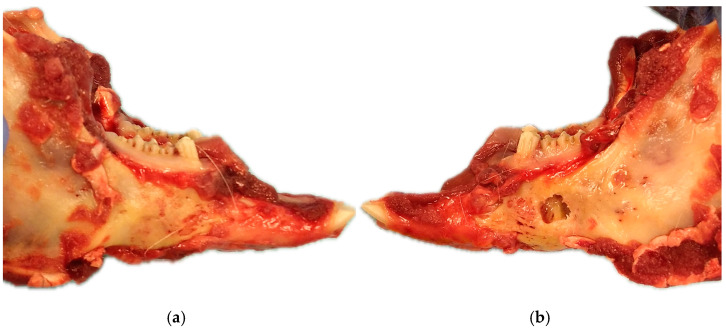
Macroscopic appearance of a bilateral view of mandible defect healing. (**a**) Right side of the mandible, where the defect was treated with a membrane, bone regeneration appears to be complete, demonstrating effective structural healing. (**b**) The left side, which was left untreated, still displays the unhealed defect, indicating the critical nature of the defect.

The Macroscopically examination revealed that none of the rabbits showed signs of infection or extrusion of the membranes. Visually, the covered area appeared to exhibit complete regeneration. In contrast, the control area maintained the created defect (Figure 12).

**Figure 13:**
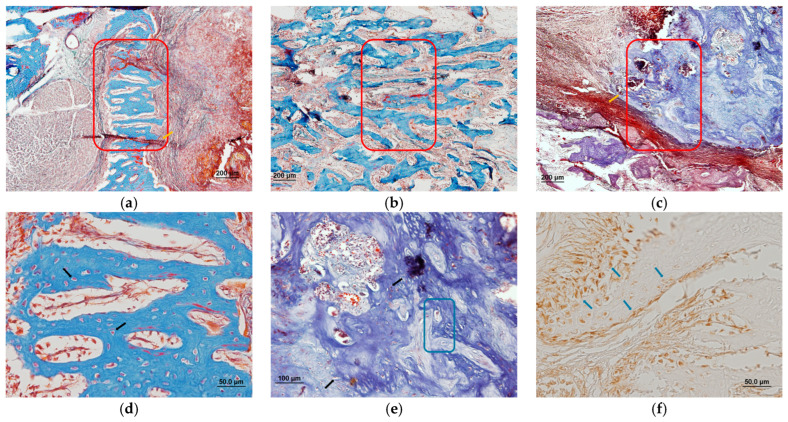
Histological analysis of covered defects observed by conventional light microscope stained with Masson’s trichrome (**a**–**e**) and Vimentin (**f**).

Histological analysis of the areas treated with the membrane demonstrates enhanced activity in specific regions, as highlighted by red squares in Figure 13a–c. These regions exhibit evidence of bone regeneration, with spongy bone trabeculae and intertrabecular spaces surrounded by connective tissue. Furthermore, reddish areas, pointed out by yellow arrows, represent highly vascularized connective tissue, indicative of ongoing remodeling—a typical feature of regenerative environments.

At higher magnification, blue-stained areas of bone matrix are evident. Red-stained *nuclei*, identified with black arrows (Figure 13d,e), correspond to osteoblasts and other cells integral to bone regeneration. Additionally, regions filled with immature osteoid tissue surrounded by extracellular matrix were noted to occupy some of the empty spaces. The immature nature of this matrix is suggested by its loose, undulating texture, as is denoted by the blue square in Figure 13e.

The cellular organization, indicated by blue arrows in Figure 13f, reveals the presence of mesenchymal cells and fibroblasts. Their spatial arrangement suggests a coordinated effort to form a structural scaffold, a critical precursor to the synthesis of new bone matrix in regenerating areas.

## 4. Discussion

The repair of bone defects in the craniofacial area, secondary to trauma, benign tumors, oncological resections, or congenital pathology, constitutes a complex challenge for surgical specialists. Currently, the technique considered the gold standard to repair this type of defect is the use of autologous bone grafts, where bone tissue is transplanted from distant sites within the patient’s body—such as the fibula, tibia, scapula, or iliac crest—alongside vascular microsuture techniques to ensure viability [16,17].

This method is highly effective due to its stable structure, low immunogenic response, and intrinsic osteogenic potential, which collectively facilitate optimal bone healing. Nonetheless, the harvesting procedure carries a significant complication rate of 10–40%, including hemorrhage, nerve and vascular lesions, and postoperative pain [18,19,20,21,22]. Furthermore, the limited availability of donor tissue and, ultimately, the poor efficiency of these treatments have been touching the scientific community to investigate alternative routes.

In the practice of GBR, clinicians often employ biomaterials as barriers to prevent the formation of fibrous tissue in the wound while enhancing the healing process [23]. Technically, the membrane serves as a barrier between the soft tissue and the bone defect area, thereby inhibiting the migration of non-osteogenic cells into the defect site and providing the osteogenic cell population of the native bone an opportunity to grow [24]. Nevertheless, numerous authors concur that in many studies, the mechanical and biological properties of the fabricated materials are not fully investigated [25,26,27,28].

For GBR, collagen membranes stand out as particularly promising biomaterials as they offer attractive biological signals that promote cell and tissue integration in vivo with a reduced inflammatory response, and, in addition, they represent biodegradation and bioresorption capabilities in which gradual absorption aligns with tissue regeneration needs [25,29].

Compared with the nonabsorbable membranes, the absorbable membranes show less immunogenicity and, therefore, decrease the risk of mucosal dehiscence and inflammation [25,27,30]. In addition, some absorbable membranes have immunomodulatory effects because of their specific crosslinking manner. For instance, the collagen matrix, an extracellular matrix, and soluble signaling molecules contained in the epugallocatechin-3-gallate crosslinked collagen absorbable membrane can significantly downregulate the inflammatory response in local tissues, recruit M2 macrophages, and accelerate wound healing [31].

The biological properties of collagen membranes vary significantly depending on the source and structure of the collagen. Within the “collagen world”, mammalian origin collagens still occupy the leader’s position. Commercially available resorbable collagen membranes are typically derived from porcine or bovine dermis or bovine pericardium. Furthermore, the risk of disease transmission to humans, such as bovine spongiform encephalopathy, and the associated religious constraints, together with ethical perspectives refraining from the use of materials from mammal origin, have been questioning its use [22,23]. Therefore, to achieve desirable GBR outcomes, the biological properties of the collagen membrane and the patient’s bone healing ability need to be considered when selecting the collagen membrane. A large number of clinical data show that it takes at least three months to completely regenerate the bone in the jaw [32,33,34]. At present, it is difficult to select the collagen membrane that our patients need without scientific knowledge of its biological and mechanical properties. Ideally, the degradation rate of the collagen membrane should match the rate of bone formation [35]

The production of our collagen membranes takes a unique approach by utilizing remnants from clinically used bone grafts, ensuring the traceability of the manufactured material and its exceptional biological safety. This innovation positions our collagen membranes as a trusted, cutting-edge solution that not only fulfills modern clinical requirements but also addresses longstanding issues related to collagen sourcing.

However, the primary contribution of this work lies in its focus on the circular economy. Today, few doubt that the primary economic challenge worldwide is to mitigate the adverse effects of progressive industrialization on the environment. The economic growth associated with this trend correlates with increased production not only of consumer goods but also of specialized items that are difficult to manufacture, such as medical supplies. Measures to reduce the production of hard-to-recycle waste are becoming increasingly important to manufacturers, with the implementation of circular economy principles now a priority for the European Union [4].

Treating waste, in our case bone waste from a tissue bank, as a valuable raw material converting it into manufactured collagen membranes suitable for clinical use is our main objective. This is our idea: collagen membranes produced from the residual material of long bones (femur, humerus, and others) obtained from human cadavers, which are already used clinically as allogeneic grafts. The production process is cost-effective, can be implemented in any hospital tissue bank, and aligns with the principles of a circular economy by repurposing biological waste. This led us to our key question: Is clinical application feasible? To address this, we conducted thorough in vitro and in vivo analyses in a rabbit model, aiming to assess their potential in regenerative medicine.

The mechanical properties of biomaterials have been one of the most fundamental determinants of their suitability for biomedical use [36].

Early research identified substrate elasticity (or stiffness) as a key factor directing cell spreading and migration [37]. Subsequently, several research groups demonstrated the role of substrate stiffness on a wider range of cellular behavior, such as differentiation of mesenchymal stem cells [38,39].

The low rigidity and minimal thickness of the manufactured material in this work (“film” type) introduced an element of uncertainty in both the handling of the samples and the cutting process. Initially, it was determined to conduct the analysis using the hydrated material (saliva), as this represents the condition in which it would be managed in clinical practice. However, this option was ultimately dismissed because it compromised the repeatability of the tests by significantly increasing the difficulty of handling and positioning the samples within the testing equipment.

Additionally, it was not possible to measure the thickness of the hydrated samples using the electron microscope (SEM, JEOL JSM-5600, JEOL Ltd., Akishima, Tokyo, Japan, acquired through JEOL’s European distribution network). Ultimately, it was decided to analyze the dehydrated material. Although the unhydrated samples will exhibit different behavior compared to the hydrated ones, the results obtained allowed us to compare the performance among the different batches of collagen membranes, which proved to be acceptably homogeneous. In any case, previous research showed that a moist environment has a slight impact on the mechanical properties of the membrane [40]. We currently know that the tensile strength and Young’s modulus of the wet group are slightly lower than those of the dry group [34,41].

Initially, straight-shaft test specimens in strip format were used. However, the absence of anchor heads resulted in damage to this area’s structure when the material was clamped in the jaws of the testing machine, invalidating an accurate determination of the tensile strength. For this reason, this specimen geometry was discarded, and a bone-type specimen was designed, consisting of a central zone with a smaller cross-section and wider head areas (Figure 1). This ensured that the sample would break in the central zone due to tensile stress alone.

It is well established that the mechanical properties of absorbable membranes are worse than those of nonabsorbable membranes [33,42].

Based on the mechanical results obtained from the samples in our study, it is shown that batches 1, 2, and 3 exhibit very similar behavior, which can be considered representative of the material. Experimentally, we confirmed that the failure of the specimens occurs at a strain level very close to 3.75%, indicating the point where elastic behavior transitions to plastic (permanent deformation). Furthermore, the membranes of the present work reach a breaking stress range of 50 to 80 MPa, demonstrating substantial tensile strength. This remarkable property makes them significantly stronger than standard collagen membranes, which typically exhibit considerably lower tensile strength [34,36,43,44].

High tensile strength and optimal elasticity are crucial for collagen membranes in guided bone regeneration, as they need to withstand physiological forces without tearing or collapsing. Overall, our membrane exhibited appropriate mechanical properties. Its Young’s modulus and elongation rate observed suggests that it can maintain the space to be regenerated without using pins or screws, showing appropriate flexibility to adapt to the defect to be regenerated. On the other hand, in the literature, it has been determined that Young’s modulus values of 0.25 to 3 GPa correspond to pure type I collagen fibrils, as occurs in our membrane [45,46].

The thickness of the membranes produced ranges from 40 to 50 μm, significantly thinner than those typically used in GBR. Despite this reduced thickness, or perhaps because of it, the membranes display mechanical properties different from those of conventional membranes. The appearance of our membrane is reminiscent of a contact lens: thin, transparent, and adaptable to the defect in situ, allowing it to adhere without the need for chemical or mechanical fixation methods (such as adhesives or tacks).

Scanning electron microscopy images of our membranes show that they are primarily composed of type I collagen, which has demonstrated the ability to interact and transduce signals essential for cell migration and proliferation. These properties, together with their presence in various tissues and biological sources, make type I collagen an appropriate material for regenerative purposes. It has been used to fabricate matrices in the form of membranes, sponges, or gels and is applied in dermal repair, cartilage reconstruction, corneal reconstruction, periodontal ligament regeneration guidance, and bone repair [11,47].

The SEM image in this study shows a highly porous, intertwined network of collagen type I fibers that appears as irregularly woven strands, creating a fibrous mesh with pores between them and exhibiting a “triple-helix” morphology. This tightly interwoven arrangement of collagen provides structural support and elasticity to the membrane while allowing sufficient porosity, both of which are vital for the successful integration and function of GBR membranes in a biological setting, as according to the literature, barrier membranes with porous structures (pore size >25 μm) provide better bone formation during the initial healing period than non-porous or smaller pore sized size membranes [48].

For this study, rabbits were chosen because they allow the creation of mandibular defects of reasonable size with easy surgical access. The molar/premolar region of the rabbit mandible provides an adequate size (17 mm in length, 16 mm in height, and 6 mm in depth) for membrane placement and subsequent analysis [49,50].

Macroscopically, the 10 mm defects created and covered with the membrane appeared to exhibit complete bone regeneration, contrasting with the untreated control area, which remained unchanged. To evaluate the osteoregenerative capacity of our membrane with micro-CT, we used two controls: on the one hand, the critical defect in the contralateral hemimandible without membrane, and on the other, the native bone surrounding the defect covered with the membrane.

Radiologically, regeneration of the defect area and micro-CT quantification analysis corroborate the macroscopic findings, revealing new bone formation with a lower degree of mineralization compared to the adjacent native bone. This differential mineralization suggests a progressive healing process, indicating that while regeneration is occurring, it may still be in the early stages relative to the more mature native tissue.

Furthermore, histological evaluations reinforce the notion of effective osteogenesis, as evidenced by the presence of osteocytes and other bone-forming cells within the newly regenerated matrix. Additionally, the absence of any signs of infection or significant alterations in microstructural integrity supports the conclusion that the regenerative process is proceeding without complications. Notably, the treated area remains free of soft tissue that could otherwise impede and obstruct the process of bone regeneration.

Studies on bone regeneration in rabbit mandibles with critical-sized defects suggest that nearly complete regeneration can be achieved within 8–12 weeks when enhanced by growth factors or stem cell-laden scaffolds [51]. In comparison, collagen membranes alone support a more gradual regenerative process, facilitating natural bone turnover rather than significantly accelerating healing [52]. Given the nine-week duration of our in vivo experiment and the reported timeframe for optimal bone regeneration, full regeneration, and mineralization can reasonably be expected by the end of this period.

In the present study, the degradation time of the tested membrane was not evaluated. However, no membrane remnants were observed in the histological sections of the experimental defects 9 weeks after membrane placement. Previous experimental studies have shown that the degradation of collagen membranes may begin as early as 4 days to 4 weeks after placement [53].

Although the in vivo and in vitro behavior of the investigated membranes appears promising, several limitations of the study must be taken into consideration. Firstly, a larger number of animals sacrificed at different time points would be required to better understand the membrane’s in vivo behavior. Secondly, it would be important to investigate the membrane’s function as a coating for critical defects filled with osteoinductive/osteogenic agents. Thirdly, the degradation rate of the membrane should be more precisely defined to ensure it is not excessively rapid, allowing adjustments in its manufacturing process if necessary. In any case, at the present time, the strength of the work is focused on the use of waste material for the manufacture of a medical product that can be applied to humans at a low cost.

## 5. Conclusions

Membranes made from human bone remnants from our tissue bank are composed of collagen type I and crosslinked using UV light. They can be easily shaped, dry-fitted, or hydrated and do not require positioning on a specific side or additional fixation with glue or tacks. Their mechanical properties make them suitable for GBR. They are also low-cost and present a reduced risk of complications, such as membrane exposure and tissue damage. By 9 weeks, membrane resorption is nearly complete.

## Figures and Tables

**Table 1:** Thickness values obtained for each batch.

ID	Batch 1		Batch 2		Batch 3		Batch 4	
	Excess	Specimen	Excess	Specimen	Excess	Specimen	Excess	Specimen
Measurements [µm]	31.7	42.7	23.3	41.6	40.1	42.9	20.5	21.1
	29.4	42.4	24.8	44.7	42.9	44.0	20.0	21.1
	27.8	42.7	23.5	45.1	39.0	42.7	20.0	21.1
	29.6	42.7	23.2	45.4	37.7	42.9	20.0	20.5
	30.9	42.1	23.2	43.1	36.6	41.3	19.7	22.7
	28.7	40.5	22.1	45.8	52.3	41.1	16.0	23.0
	30.1	40.3	21.9	44.6	46.0	41.4	18.0	23.0
	28.8	41.1	22.4	44.0	45.2	39.0	17.7	22.5
	28.5	41.3	21.9	44.9	42.7	38.1	16.1	21.9
	28.0	41.3	20.5	45.4	38.4	37.6	13.7	21.6
Mean [µm]	29.4	41.7	22.7	44.5	42.1	41.1	18.2	21.9
Deviation [µm]	1.2	0.9	1.1	1.2	4.5	2.1	2.2	0.9

**Table 2:** Value of Young’s modulus and tensile strength obtained for each specimen.

	L1a	L1b	L2a	L2b	L3a	L3b	L4a	L4b	L4c
E [GPa]	2.77	2.22	2.58	2.84	2.34	2.65	2.91	2.74	2.85
σ_u_ [MPa]	71.7	57.8	66.9	80	53.3	66.4	73.5	29.1	20.1

**Table 3:** Characteristic mechanical parameters of collagen membranes (Batches 1, 2, and 3).

	Mean	Deviation
E [GPa]	2.56	0.24
σ_u_ [MPa]	65.43	9.57

**Table 4:** Descriptive and inferential values in the five variables investigated with micro-CT.

		*n*	Mean	Std. Deviation	95% Confidence Interval for Mean		*p*-Value
					Lower Bound	Upper Bound	
Mean BMD	Native bone	4	1.11	0.10	0.94	1.28	<0.05
	Cortical bone	4	0.99	0.09	0.83	1.14	
	Trabecular bone	4	0.23	0.10	0.06	0.39	
BV/TV	Native bone	4	70.22	4.72	62.70	77.74	<0.05
	Cortical bone	4	70.00	11.55	51.60	88.39	
	Trabecular bone	4	8.23	5.38	−0.32	16.80	
Tb.Th	Native bone	4	0.11	0.01	0.09	0.14	n.s
	Cortical bone	4	0.14	0.03	0.08	0.20	
	Trabecular bone	4	0.10	0.01	0.08	0.12	
Tb.Sp	Native bone	4	0.08	0.01	0.06	0.10	<0.05
	Cortical bone	4	0.11	0.02	0.07	0.15	
	Trabecular bone	4	1.33	0.87	−0.05	2.73	
Tb.N	Native bone	4	6.04	0.36	5.45	6.62	<0.05
	Cortical bone	4	5.09	0.74	3.91	6.27	
	Trabecular bone	4	0.80	0.48	0.03	1.57	

BMD, Bone Mineral Density; BV/TV, Bone Volume/Total Volume; Tb.Th, Trabecular Thickness; Tb.Sp, Trabecular Separation; Tb.N, Trabecular Number; n.s., not specified.

## Data Availability

Datasets available in this study are available on request from the corresponding author.
